# Environmental perception of gatherers of the crab 'caranguejo-uçá' (*Ucides cordatus*, Decapoda, Brachyura) affecting their collection attitudes

**DOI:** 10.1186/1746-4269-1-10

**Published:** 2005-11-03

**Authors:** Rômulo RN Alves, Alberto K Nishida, Malva IM Hernández

**Affiliations:** 1 Departamento de Biologia, Universidade Estadual da Paraíba and Programa de Pós-Graduação em Ciências Biológicas (Zoologia), Departamento de Sistemática e Ecologia, Universidade Federal da Paraíba, 58051-900 João Pessoa, PB, Brazil; 2 Departamento de Sistemática e Ecologia, Universidade Federal da Paraíba, 58051-900 João Pessoa, PB, Brazil, PB, Brasil

## Introduction

Mangrove forests are productive ecosystems found along the coastal zones of Brazil, providing several profitable resources such as timber, medicinal products, natural dye, fish, crustaceans, and molluscs. For the littoral dwellers of Northeast Brazil, the Brachyura crabs are a major economic resource. The main species they commercialize are the 'goiamum' (*Cardisoma guanhumi*), 'siris' (*Callinectes *spp), and the land crab 'caranguejo-uçá' (*Ucides cordatus*). The land crab is the most exploited species, and of most relevance for people living in the surrounding mangrove areas in the State of Paraiba [1-3].

*U. cordatus *lives in an individual burrow *ca*. 1 m deep, situated under mangrove trees. Adult crabs have few predators, notably the crab-eating racoon (*Procyon cancrivorous*), monkeys, and hawks [4]. Despite this, a high predation pressure on *U. cordatus *is exerted by humans who harvest this species for food [5]. In the Northeast Region of Brazil, the exploitation of *U. cordatus *holds particular socio-economic importance since it involves many local residents, who benefit from both direct and indirect employment [2,3]. Crab gatherers have observed that the natural stock of *U. cordatus *has decreased alarmingly since 1998, when an unexpected crab mass-mortality event occurred in the mangrove habitats of the Paraiban littoral [3]. The subsequent low crab abundance created social problems in the surroundings of those mangrove areas and seriously affected the economic welfare of poor people who depend upon crab gathering for their livelihoods.

The need for research on the exploitation of mangrove ecosystems and on *U. cordatus *in particular was emphasized by Maneschy [6], who also suggested the need to study the socio-economics of crab collecting, which has recently experienced an increase in production demand. The life of gatherers is intimately linked to ecological processes and cycles, and their daily involvement with the exploitation of other natural resources will likely help them to develop harvesting strategies for maximizing the crab catch efficiency. An understanding of the ecology of *U. cordatus *by local gatherers is an important component of the process of exploitation [2,6].

In recent years, researchers have emphasized the importance of traditional knowledge amongst fishermen. They have also emphasized the potential role that traditional fishing practices can play in the development and implementation of sustainable fishery management in the modern world [7-9]. Human communities which rely directly on their natural resources for subsistence, often have a detailed understanding of their local environment [10-12]. The economic, social, and cultural activities of such people often depend upon local environmental goods and services [13].

Ecologists and environmental managers have generally disregarded the possibilities of learning from the traditional human communities [14]. However, a recent acknowledgment of their relevance has led to an intensification of studies on traditional knowledge [14-18]. In Brazil alone, Diegues [19] listed 868 relevant publications on traditional human populations, of which nearly 80% were published over the last 20 years, and mainly in the last decade.

Traditional knowledge may help in the establishment of management plans aimed at the sustainable exploitation of natural resources [2,3]. Nordi [20] observed that the government environmental organization controlling the capture of *U. cordatus *does not consider local human knowledge of the ecology of the species, a fact that possibly explains the poor effectiveness of the regulations governing the exploitation of this resource. *U. cordatus *individuals are caught manually or by the use of some tools which allow easier access to them. In most of Brazilian States professional crab gatherers are male [21].

During normal harvest procedures gatherers select crabs with respect to both sex and size. In particular, male crabs are preferred due to their higher flesh yield [22]. Gatherers have developed the ability for distinguishing the sex of crabs as inferred from the track the animal leaves close to burrow openings, as well as from the size of burrow entrances. This perception is important since it directly influences the capture process since large male crabs are preferred due to their higher commercial value. The aim of the present work is to evaluate the ability of gatherers to discriminate the sex and size of crabs, and the importance of this ability on the development of successful harvest strategies. It is also intended here to evaluate the implications of this perception for establishing measures aimed at the conservation and management of *U. cordatus*.

## Methods

### Study areas

**Figure 1:**
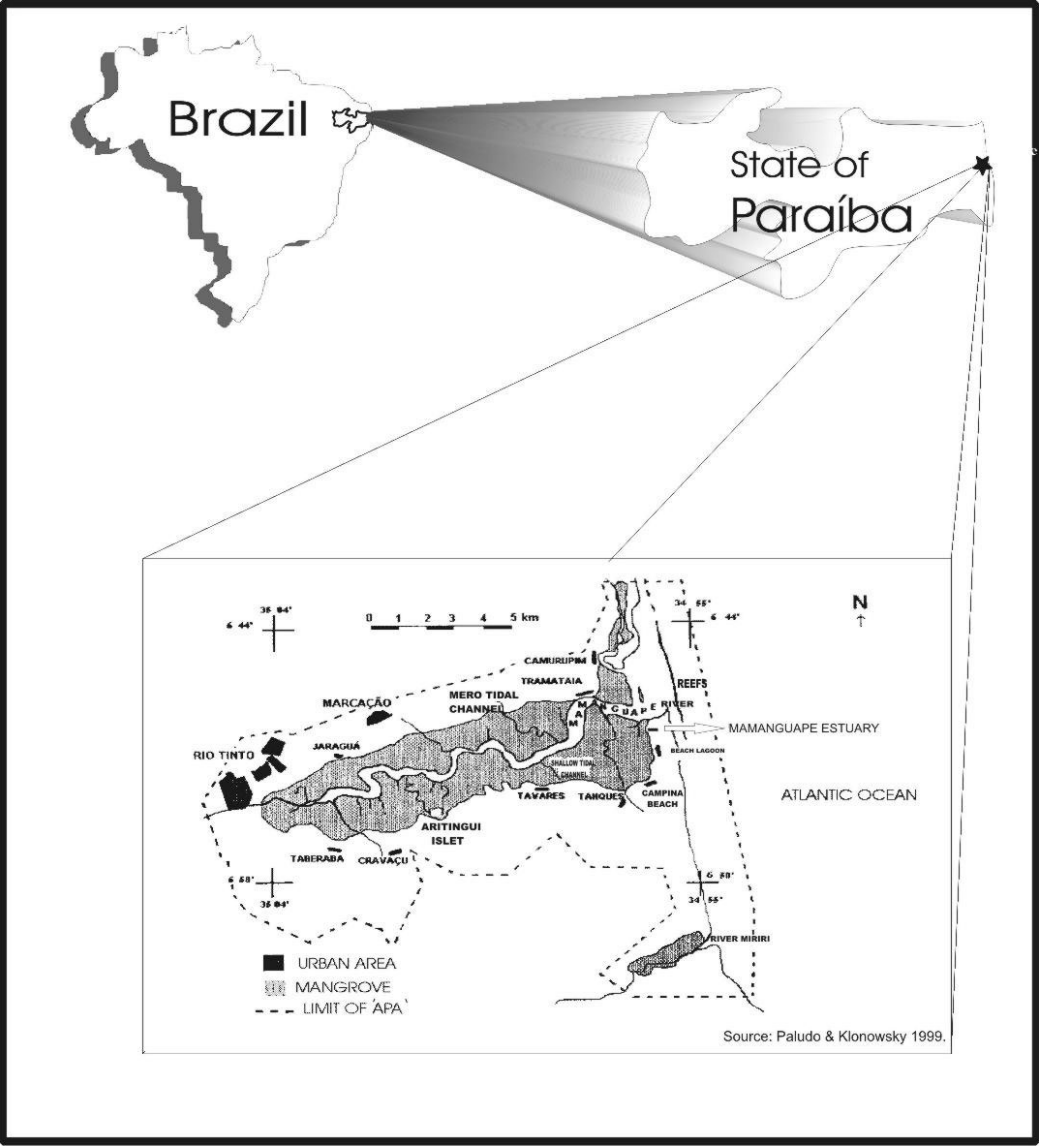
Map showing the estuary of the River Mamanguape.

**Figure 2:**
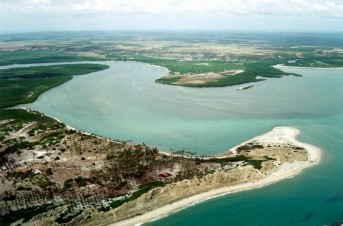
Aerial view of River Mamanguape estuary (Photo: João Carlos).

The research was carried out in the mangrove forests of the Mamanguape river, Northeast Brazil, located between lat 06°43' and 06°51' S, and long 35°07' and 34°54' W. The estuary studied here, is nearly 24 km long from east to west, and 2.5 km wide nearest to the river mouth (Figs 1 and 2).

All the area of the influence of the Mamanguape river is within the permanent protected area (APA, in Portuguese) of 'Barra do Rio Mamanguape' of IBAMA (the Brazilian Institute for the Environment and Natural Resources), formed also by the estuaries of the rivers Miriri and Estivas, and covering a total of 14,460 ha. In that estuary there is a large extension of mangrove forest, islands, and islets (the latter formed by sandy-clay-loam banks), and barrier reefs in the rivers mouths together form an elongated sand bar. The whole area is located in the municipalities of Rio Tinto, Marcação, and Lucena, in the State of Paraíba (Figure 2).

The estuary of the APA includes *ca*. 6,000 ha of a reasonably well preserved mangrove forest, the largest of its kind in the State of Paraíba. The common trees are *Rhizophora mangle*, *Avicennia germinans*, *A. schaueriana*, *Laguncularia racemosa *and *Conocarpus erectus*. The tallest *R. mangle *trees reach 25 m and are up to 60 cm DBH (diameter at breast height); *A. germinans *trees are taller than 30 m and have up to 65 cm DBH [1].

Despite the fact that the Mamanguape mangrove forest is relatively well preserved, some sites appear to be affected by anthropogenic activities, mainly derived from sugar cane cultivation. Watanabe [23] reported water contamination in one of the estuary tributaries from sugar cane plantations. According to local fishermen, a decrease in fish production has been observed due to an increase in the amount of agrochemicals used in plantations along the river banks.

### Procedures

The research was performed between November and October 2002. Before starting the field work, a meeting was held with crab gatherers to inform them about the goals of our study, and research methods, and to propose their participation in our investigations.

In order to respect indigenous intellectual property rights, we followed an informal research protocol: before the survey, we briefly explained the nature of the research and specific objectives to each interviewee [28]. We were precluded from adopting a formal approach using interview consent forms, owing to the poor level of organization among the community of gatherers, and high illiteracy rates [2,3].

**Figure 3:**
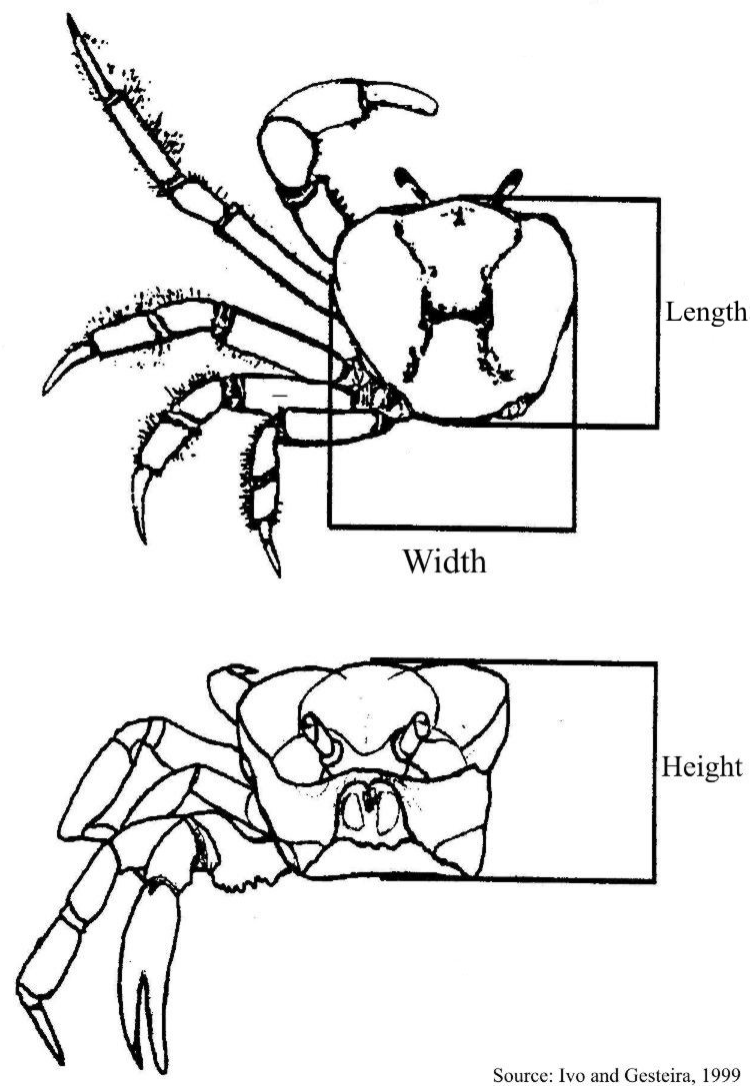
Morphometric measurements taken from the carapace of *Ucides cordatus *(Photo: Guy Nishida).

We selected ten gatherers typical of the community of gatherers, with each having at least 20 years of experience and being from the middle income range. The perception and discrimination ability of crab gatherers was analysed through direct observations, as a 'non-member participant observer' [24], complemented by open interviews and informal conversations [25-27]. Each gatherer was interviewed individually, there was no time limit for the interviews and they lasted between 1 and 3 hours each. We joined the gatherers (one at a time) in their daily activities. During the course of the study they captured 210 crabs. Morphological measurements were taken using venier callipers, accurate to 1 mm. Crab carapaces were measured with respect to: (a) height, taken dorsoventrally from its central point; (b) length, taken from the sagittal plain on the dorsal surface of the animal; and (c) width, taken transversally from the first pair of pereiopods. The carapace width corresponds to the largest body dimension (Fig. 3). The burrow opening of each caught animal was simultaneously measured with respect to height (the longest axis) and to width (the shortest axis).

The gatherer was asked to guess the sex the animal he was going to catch. His perception was then evaluated after a careful examination of the crab external sexual characteristics, following Mota Alves [29]. The possible association between what gatherer expectation and observed sex classification was evaluated using a chi-square test in a 2 × 2 contingency table with Yates correction [30].

## Results

The carapaces length ranged from 3.2 cm to 5.8 cm, with a mean of 4.27 cm (± 0.424); the width ranged from 4.1 cm to 6.7 cm with a mean of 5.53 cm (± 0.537); and the height ranged from 2.6 cm to 6.2 cm with a mean of 3.52 cm (± 0.480). The height of the burrow openings ranged from 3.4 cm to 7.3 cm with a mean of 5.72 cm (± 0.694), and the width ranged from 3.2 cm to 7.1 cm with a mean of 5.21 cm (± 0.693). The data for both carapace size and burrow entrance size were normally distributed.

The correlation analysis performed on the burrows opening dimensions and carapace dimensions showed positive and significant correlations (p < 0.05) (Table 1).

**Table 1:** Pearson's correlation (*r *values) between the burrow opening dimensions and the *Ucides cordatus *carapace dimensions (n = 210; * significant at p < 0.05).

Burrow opening dimensions (cm)	Carapace dimensions (cm)		
	Carapace height	Carapace length	Carapace width
Burrow opening height	0.37*	0.58*	0.59*
Burrow opening width	0.40*	0.60*	0.62*

The variables with the most biological significance with respect to crab behaviour of going into and coming out of the burrows, are the width of the burrow opening and the crab carapace length. The regression analysis performed with these two variables generated the equation: *burrow opening width = 1.02 + 0.98 × crab length*; and showed that each centimetre increase of the carapace length corresponds to 0.98 cm increase of the burrow opening width (r = 0.60; p < 0.05).

**Figure 4:**
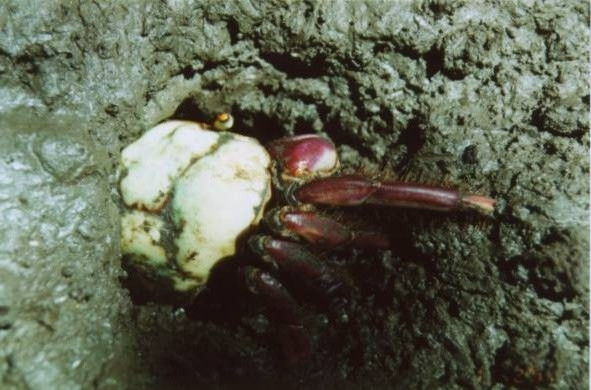
*Ucides cordatus *specimen entering the burrow (Photo: Guy Nishida).

**Figure 5:**
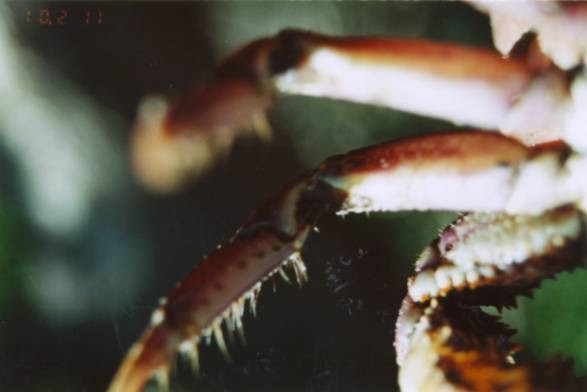
Legs of *Ucides cordatus *females virtually without hairs (Photo: Guy Nishida).

**Figure 6:**
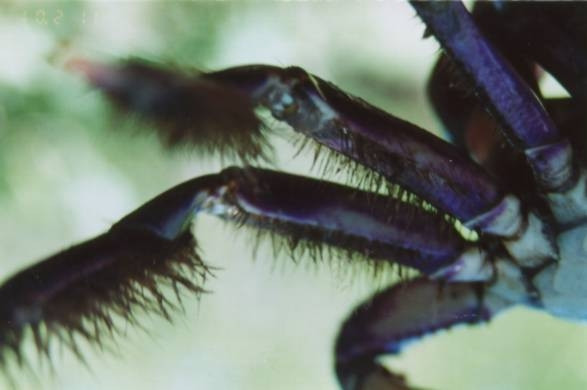
Hairy legs of *Ucides cordatus *males (Photo: Guy Nishida).

**Figure 7:**
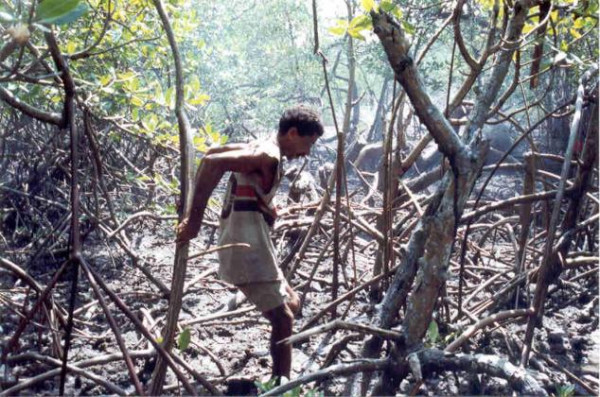
Gatherer of the land crab 'caranguejo uçá' (*Ucides cordatus*) in the mangrove habitat the River Mamanguape estuary.

The crab gatherers reported that they are able to distinguish male from female animals through the tracks their limbs leave on soil at the burrow entrance (Fig. 4). The females do not have virtually any hairs in their legs (Fig. 5), and leave thin deep tracks at the burrow entrance, in opposition to the hairy legs of males (Fig. 6), leaving relatively wide and shallow tracks. Figure 7 shows a land-crab gatherer during harvesting activities.

The data obtained in this study showed a highly successful perception of crab sex amongst gatherers, i.e. 75.2% of the 210 individuals caught were successfully identified with respect to sex, of which 45.2% were males and 30% were females (Table 2). The chi-square test showed a positive and highly significant association (χ^2 ^= 53.27; df = 1; p < 0.01) between the animal sex expected by the crab gatherer, and the sex recorded following examination of the animals. The 24.8% of mis-identifications comprised of 6.7% of the males and 18.1% of the females they caught. Consequently, mis-identifications are nearly three times more likely in the case of female individuals compared to males (Table 2).

**Table 2:** Percentage success of sex perception of *Ucides cordatus *crabs by local gatherers.

Sex expected by the gatherer	Sex observed		Gatherer hits (%)	Gatherer misses (%)
			
	Male	Female		
Male	95 (hit)	38 (miss)	45.2	18.1
Female	14 (miss)	63 (hit)	30.0	6.7
Total	109 (51.90%)	101 (48.10%)	75.2	24.8

## Discussion

The proportionality between the crab's carapaces and the burrow openings had already been reported by other authors [31-33]. Their results were confirmed by this present study.

The correlation analysis showed that the crab morphometric values of length and width, as well as burrow opening dimensions were the main variables which had a significant association. The following conclusions can be reached from our results: carapace height was weakly correlated with burrow opening height (r = 0.37) and with burrow opening width (r = 0.40), probably because *U. cordatus *individuals enter the burrow by flexing their chelipeds frontally to the body, in a way that the longest axis of the burrow opening (its height) corresponds to the carapace height (Figure 3). This lateral movement of young and adult male and female crabs was also observed by Costa [33]. Geraldes and Calventi [34] reported that the 'caranguejo-uçá' enters the burrow laterally, positioning first either the largest or the smallest claw. They also observed that the burrow opening was a little larger (0.34 cm) than the crab carapace length.

The gatherers' observation that the larger the burrow the bigger the crab (as was confirmed by the present study), is important with regard to maximising the harvest obtained by the gatherers.

With respect to the gatherers perception of crab sex, Maneschy [6] also reported that in Pará State, North of Brazil, they distinguish the crabs' sex through their tracks left in front of the burrow openings, but that sometimes mistakes are made. According to crab gatherers of River Mamanguape area, some other factors affect their ability to successfully discriminate the sex of crabs, including the tide dynamics and sediment compaction. Areas washed away by high tides or with hardened sediments, are difficult places to recognize the crabs tracks. Gatherers are also aware of the sex and size of the crabs they target by direct observation of the burrow entrances. Therefore the catches are selective, since large male specimens have a higher commercial value.

Based on the catch selectivity of *U. cordatus *individuals, where both smaller sized and female individuals are not collected but returned to their habitat, Ivo and Gesteira [22] stated that this crustacean can be sustainably harvested. However, despite this conclusion, depletions of natural stocks of *U. cordatus *have been reported in the States of Paraíba [2,3], Espírito Santo [35], and Pará [36]. It is therefore important to consider several other factors, besides exploitation through human harvesting, that have contributed to reduce natural populations of *U. cordatus*, the most important of which is habitat degradation. In Brazil, like in many other tropical countries, mangrove forests have been widely cleared through anthropogenic activities

Another important factor is the precarious social conditions within which the crab gatherers' families survive [2,3], a factor that is associated with the unpredictability of both the capture success of the animal and market demand, and often forces gatherers to harvest crabs in a non-selective approach [37]. All interviewees had a low income of about US$ 100 per month (roughly equal to the minimal wage in Brazil). Crab gathering is the most important means of subsistence for the families of the crab gatherers, despite their supplemental income from small-scale agriculture activity, and the extraction of other natural resources. Crab gatherers are economically marginal groups, extremely poor and largely ignored by other artisanal fishermen. Despite this, the animals they capture are an important component of regional cuisine, as well as being sold commercially in urban centers.

The focus on crab harvesting as providing the economic foundation of these communities may lead them to an economic dependence on buyers, who are therefore in a position to exploit the gatherers. This would mask their perception about their harmful actions on the environment. In the case of common resources, a subject discussed by Burke [38], we agree that '... simply because resource users are not aware of the collective environmental costs of resource use does not make them irrational. It simply means their resource use follows a rationale other than the logic of the commons – possibly the logic of consuming more of a thing for which they have a preference'.

At the present time and despite prohibitions imposed by the government concerning the minimum size of crabs allowed to be caught, small sized and female specimens are also being collected. Therefore, the size-selective harvesting is practised only at sites where large crabs are abundant. This present situation may lead to an over-exploitation of crab populations in mangrove forests of the State of Paraíba, especially at sites presently experiencing strong anthropocentric pressure. A similar situation has been observed in the mangrove ecosystems of the States of Rio de Janeiro and São Paulo, where *U. cordatus *is considered to be under risk of extinction [39,40].

As observed by Bergallo [39] the decrease in the rate at which the individuals of some species can meet each other, is a clear sign of the reduction in population size. This observation corroborates the need to carry out conservation programs, especially considering that *U. cordatus *is a valuable economic resource. The strong (and perhaps unsustainable) predation pressure exerted on this species through human exploitation can be interpreted as a consequence of *(1) *the lack of an efficient environmental management policy, *(2) *the lack of studies and actions regulating the commercialization of its catch, and *(3) *the lack of alternative socio-economic proposals aimed at improving the welfare of local human communities directly involved with the exploitation of *U. cordatus*.

The implementation of a successful management strategy fundamentally requires the involvement of the main stakeholders, who must be made aware of the need for the conservation of the natural resource as a guarantee for its sustainable exploitation [41]. In this sense, the gatherers are critical stakeholders in the process of establishing management plans, which would ensure the sustainable exploitation of the resource. In Brazil, management of coastal fisheries is usually imposed on local communities by the federal government, without taking the either traditional knowledge or the reality of their socioeconomic conditions into consideration – with negative consequences for management plans.

The knowledge derived from traditional fishermen may contribute to strategic ecological managements [42], particularly in research areas with scarce or non-existent data [43,44]. Ethnoecological studies may also help in promoting dialogue and cooperation between fishers and scientists. The scientific literature illustrates the significance and value of traditional knowledge in Brazil. Alves and Nishida [2], for example, reported on the influence of tide variations on the ecdysis of the mangrove crab *Ucides cordatus *in natural environments, based on information obtained from gatherers. Marques [45] also reported the gathering of relevant local information on the diet of an estuarine fish (*Arius herzbergii*, Ariidae).

Cultural values and perceptions arising from more experienced elder people need to be taken into account before any decision about their livelihoods can be considered by the relevant governmental authorities. Their contributions are essentially concerned with the sustainable management of local resource, and such contributions unfortunately have not yet been given satisfactory representation in the decision making process.

## Conclusion

The results obtained here make evident that gatherers of *Ucides cordatus *have developed an intimate knowledge of the natural history of this species. Thus, they have developed skills that led them to become efficient harvesters. The unique nature of this local knowledge demonstrates the need for considering these factors in the implementation of management plans of coastal mangrove ecosystems. Gatherers' knowledge can provide a useful basis for understanding local crab stocks and their population dynamics. This kind of information may be used for establishing extractive reserves as well as for delimiting the harvest season and for establishing protected areas where animal species reproduce, aiming to maintain their natural stocks.

In the State of Paraíba the law prohibits that females of any size and males smaller than 4.5 cm of carapace length be captured. We believe that the re-examination of the existing laws of crab harvesting and other crustaceans are urgently needed for the preservation of natural stocks of these animals. The gatherer's environmental perception and its consequent influence on capture efficiency, are the main factors to be considered when making decisions about sustainable exploitation of mangrove ecosystems resources. Such decisions would only be effectively applied if the local knowledge held by crab gatherers as well as their socioeconomic conditions do not continue to be ignored by governmental authorities.
